# Biochemical markers of depression - an up-to-date review

**DOI:** 10.1192/j.eurpsy.2021.1833

**Published:** 2021-08-13

**Authors:** A. Nobis, D. Zalewski, E. Dąbrowska, N. Waszkiewicz

**Affiliations:** Department Of Psychiatry, Medical University of Białystok, Choroszcz, Poland

**Keywords:** inflammatory, Depression, biomarkers, oxidative stress

## Abstract

**Introduction:**

Depression or Major Depressive Disorder (MDD) is the most prevalent psychiatric disorder and a leading cause of disability worldwide. Currently affecting around 300 million people worldwide, depression is a major clinical, emotional, and socioeconomic strain for society. There is a growing interest in the biological underpinnings of depression, which are reflected by altered levels of biomarkers.

**Objectives:**

The aim of the study was to present an up-to-date review of potential MDD biomarkers.

**Methods:**

PubMed, Scopus, and Web of Science databases were searched.

**Results:**

Enhanced inflammation has been reported in MDD, as reflected by increased concentrations of inflammatory markers – interleukin-6, C-reactive protein, tumor necrosis factor-α, and soluble interleukin-2 receptor. Dysregulation of the hypothalamus-pituitary-adrenals axis, along with increased cortisol levels, have also been reported in MDD. Oxidative and nitrosative stress also plays an important role in the pathophysiology of MDD. Notably, increased levels of lipid peroxidation markers are characteristic of MDD. Kynurenine metabolites, increased glutamate and decreased total cholesterol are also features of MDD. Finally, alterations in growth factors, with a significant decrease in brain-derived neurotrophic factor and an increase in fibroblast growth factor-2 and insulin-like growth factor-1 concentrations have also been found in MDD.

**Figure:**
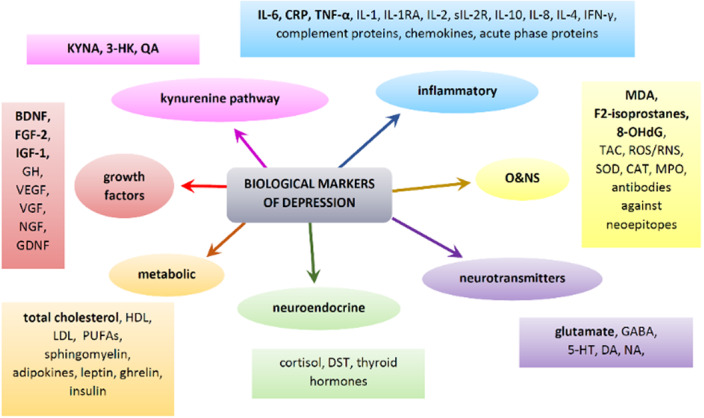

**Conclusions:**

A group of substances holds promise as reliable biomarkers for MDD. However, biomarker research in depression faces many difficulties, such as insufficient understanding of MDD etiopathogenesis, substantial heterogeneity of the disorder and low specificity of biomarkers. The construction of biomarker panels and their evaluation with use of new technologies may have the potential to overcome the above mentioned obstacles.

**Disclosure:**

No significant relationships.

